# Genome Sequence of *Cobetia* sp. Strain MM1IDA2H-1, a Hydrocarbon-Degrading and Biosurfactant-Producing Marine Bacterium

**DOI:** 10.1128/genomeA.00132-17

**Published:** 2017-04-13

**Authors:** Claudia Ibacache-Quiroga, Christian Canales, Mariam Charifeh, M. Alejandro Dinamarca

**Affiliations:** a Centro de Micro-Bioinnovación (CMBi), Universidad de Valparaíso, Valparaíso, Chile; b Centro de Biotecnología (CEBUSS), Facultad de Ingeniería y Tecnología, Universidad San Sebastián, Concepción, Chile; c Escuela de Nutrición y Dietética, Facultad de Farmacia, Universidad de Valparaíso, Valparaíso, Chile

## Abstract

*Cobetia* sp. strain MM1IDA2H-1 is a marine bacterium isolated from seawater samples that uses the heterocyclic aromatic hydrocarbon dibenzothiophene as the sole carbon source and produces a biosurfactant that inhibits bacterial quorum sensing. The *Cobetia* sp. MM1IDA2H-1 genome was sequenced, processed, assembled, and annotated for basic and applied studies.

## GENOME ANNOUNCEMENT

The genus *Cobetia* (previously classified as *Arthrobacter marinus*, *Pseudomonas marina*, *Deleya marina*, and *Halomonas marina*) belongs to the *Oceanospirillales* order, and exhibits a wide geographical distribution with remarkable eco-physiological differences and a growing number of species (1). *Cobetia* sp. strain MM1IDA2H-1 (=CECT 7764) is a hydrocarbon-degrading marine bacterium isolated from seawater samples obtained from a eulitoral pond of the Pacific Ocean (32.957165 S, 71.549770 W). During its growth with sulfur-containing heterocyclic aromatic hydrocarbon dibenzothiophene as the sole carbon source, strain MM1IDA2H-1 produces a hydroxyl fatty acid biosurfactant that inhibits quorum sensing of bacterial competitors (2). This marine bacterium is a straight aerobic Gram-negative rod, that does not grow in the absence of sodium.

The genome of *Cobetia* sp. strain MM1IDA2H-1 was sequenced in the Madrid Science Park (Madrid, Spain) using an Illumina MiSeq sequencer (System Illumina). Sequence quality was analyzed using the Prinseq tool (3). Right trimming was performed for sequence with quality < Q30, while the first base was trimmed off at the left end. Filtered sequences were assembled with SPADES (4) using pair-end and mate-pair libraries. Final assembly was evaluated with QUAST (5). Genome assemblies were annotated with the BG7 tool (6), using gene and protein sequences from the *Halomonadaceae* family as reference, which included 62,441 protein sequences from Uniprot. Aditionally, an automatic gene annotation was carried out by the Rapid Annotations using Subsystems Technology (RAST) server (7).

The genome of *Cobetia* sp. MM1IDA2H-1 has a length of 4,153,632 bp with a G+C content of 62.18% and 92 contigs (>200 bp). The *N*_50_ contig size is 730,881 bp. According to the BG7 tool, the *Cobetia* sp. MM1IDA2H-1 genome contains 3,054 annotated genes, 68 pseudogenes, and 73 RNAs (65 tRNAs, six rRNAs, and two noncoding RNAs [ncRNAs]). *Cobetia* sp. MM1IDA2H-1 genome annotation revealed the existence of peripheral and central pathways for aromatic hydrocarbon degradation, genes for solvents tolerance, gene clusters for biosynthesis of biosurfactants, and genes for resistance to copper, lead, arsenic, and mercury.

### Accession number(s).

This whole-genome shotgun project has been deposited at European Nucleotide Archive (ENA) in FASTA format under ENA accession numbers FPLA01000001 to FPLA01000105, ENA Assembly name ADCOB, ENA Study ID PRJEB17997, and ENA sample ID ERS1452906. The version described in this paper is the first version.
